# Safety and tolerability of Meningococcus B vaccine in patients with chronical medical conditions (CMC)

**DOI:** 10.1186/s13052-019-0730-y

**Published:** 2019-10-30

**Authors:** L. Nicolosi, C. Rizzo, G. Castelli Gattinara, N. Mirante, E. Bellelli, C. Bianchini, V. Pansini, A. Villani

**Affiliations:** 1 UOC Paediatrics and Infectious Disease, Bambino Gesù Children’s Hospital, IRCCS, Piazza Sant’Onofrio 4, 00165 Rome, Italy; 2 Innovation and clinical pathways Unit, Bambino Gesù Children’s Hospital, IRCCS, Rome, Italy; 3 Vaccination Center, Bambino Gesù Children’s Hospital, IRCCS, Rome, Italy

**Keywords:** Invasive meningococcal disease, Vaccination, Vaccine adverse events, Group B meningococcus

## Abstract

**Background:**

Invasive meningococcal disease is a serious global health threat in the world; in 2016, the European Centre for Disease Control and Prevention reported 3280 confirmed cases (including 304 deaths) of Invasive Meningococcal Diseases in Europe. In Italy, in 2017 were reported 200 cases 41% of which due to menB serogroup. From January 2013 the European Medicines Agency (EMA) has authorized the marketing of the meningococcal B vaccine 4CMenB.

**Methods:**

The study aimed to evaluate and complement the safety profile of 4CMenB in high risk children accessing the vaccine service of the Bambino Gesù Children’s Hospital. All individuals aged six weeks or more receiving the meningococcal 4CMenB (Bexsero®) vaccine that approached the vaccine Centre at the Bambino Gesù Children’s Hospital in Rome, were asked to participate. All parents or caregivers of vaccinated individuals in the study period, were recruited and requested to answer to a questionnaire on adverse events following immunization (AEFI) observed after 7 days, starting from the date of vaccination.

**Results:**

During the study period (October 2016–October 2017), we collected 157 completed questionnaires (out of 200 distributed). Of those 132 were first doses and 25 were booster administered doses. The median age of the study population was 4.5 years (range 0.29 to 26.8 years), the majority of subjects were high-risk individuals (64%) with chronic health conditions. Overall, 311 adverse events were reported in the 7 days after vaccine administration. In particular 147 events (47%) after administration of first dose and 58 (19%) after the booster doses. A large majority of those events, were of little clinical importance and concentrated in the 24 h after vaccine administration. No hospitalizations or Emergency Department access were reported.

**Conclusions:**

Results of our study demonstrated that the Bexsero® vaccine is almost well tolerated, with a low incidence of severe AEFIs. Our results also shown that the occurrence of AEFIs is similar within healthy and high risk children.

## Introduction

Invasive meningococcal disease is a serious global health threat in the world and has an high mortality and morbidity rate; about one in ten survivors will have major physical or neurological disabilities and Meningitis B accounts for about 80% of cases of invasive meningococcal disease in high-income countries [1]. It is the leading cause of infant bacterial meningitis and severe sepsis in Europe, and the main infectious cause of death in infancy [2]. In 2016, the European Centre for Disease Control and Prevention reported 3280 confirmed cases (including 304 deaths) of Invasive Meningococcal Diseases (IMD) in Europe, corresponding to an annual incidence rate of 0.6 cases per 100,000 inhabitants. Of these cases, 54% were caused by a serogroup B meningococcus (MenB) [3].

In Italy, in 2017, 200 cases of IMD were reported (41% of which were MenB serogroup) corresponding to an incidence of approximately 0.3 cases per 100,000 [4]. In children younger than 1 year the IMD incidence was 10 times higher (3.6/100,000).

From January 2013 the European Medicines Agency (EMA) has authorized the marketing of the meningococcal B vaccine 4CMenB (Bexsero®). The Italian National Immunization Plan (NIP) 2017–2019, [5] approved by the Ministry of Health in February 2017, introduced in the national schedule the MenB vaccination in new-borns (i.e. three initial doses at the ages of three, four and six months, with a fourth dose at the age of 13 months and infants aged > 6 months should receive three doses, with the first two given at seven and nine months of age).

The 4CMenB vaccine was licensed with a good reactogenicity and safety profile, the main adverse events following immunization (AEFI) reported in children were local tenderness/swelling or erythema and fever (14 to 50% of vaccinated) with higher figures reported when the vaccine was co-administered with other vaccines [6–11]. All this data were collected from healthy children, adolescents and adults regardless however of the clear indication of this vaccine in particular situations (e.g. anatomic and functional asplenia, and others chronic conditions). Very few study still reported data on safety on individual with chronic conditions [12].

We aimed to evaluate and complement the safety profile of 4CMenB including the use in children in special situations accessing the vaccine service of the Bambino Gesù Children’s Hospital.

## Materials and methods

### Study setting and population

All individuals aged six weeks or more receiving the meningococcal 4CMenB (Bexsero®) vaccine that approached the vaccine centre at the Bambino Gesù Children’s Hospital in Rome, between October 2016 and September 2017, were asked to participate. Eligible participants were the children who’s parents provided signed informed consent and who agreed to be interviewed by telephone or to fill in a web-based questionnaire 7 days after vaccination. Therefore, the data collection was concluded on the end of the first week of October 2017.

We excluded children with any infection within 7 days or fever within 1 day of enrollment, or administration of any other vaccine within 30 days of enrollment.

### Data collection

All parents or caregivers of vaccinated individuals in the study period were requested to answer to a questionnaire about AEFIs observed in a period of 7 days starting from the vaccination date.

We divided AEFIs in:

- local reactions: swelling/tenderness and erythema, refusal to move the extremity
- systemic reactions: unusual crying, fever
- severe adverse reactions: hypotonia, sleepiness, urticaria/angioedema

In the questionnaire parents or caregivers were requested to report solicited injection site (tenderness/swelling/erythema, refusal to move the extremity) and systemic reactions (fever, unusual crying, hypotonia, sleepiness, urticaria or angioedema), recording each AEFIs as absent, mild or severe, respectively, based on the parents’ perceived severity of AEFIs.

### 4CMenB vaccine administration

In accordance to the Novartis guidelines (recommendations), at the time of the study, the vaccine has been administered intramuscularly into the anterolateral aspect of the thigh in infants and into the deltoid muscle in children. Separate injection sites have been used if the vaccine was co-administered with other vaccines. According with Manufacture’s guideline, at the time of the study [13], infants and young children aged < 2 years were administered antipyretic agents to reduce the incidence of AEFIs (such as febrile reactions, irritability, sleepiness and poor appetite). Consequently, prophylactic paracetamol at the time of vaccination with two further doses given 4–6 h apart was recommended for all infants receiving 4CMenB along with their routine primary immunizations [14].

### Data analysis

The frequency of reported AEFIs was computed as the ratio between vaccinated individuals who reported at least one event and the total number of vaccinated individuals.

The association between the characteristics of participants and the occurrence of at least one event during the 7 days of follow-up was analyzed through a Chi-square test for categorical variables. The multivariate logistic regression model was performed taking into account all potential confounding factors available from clinical practice. In the final model, we included all variables with *p* < 0.05 in the univariate analysis, STATA software was used for the statistical analyses.

## Results

During the study period, we collected 157 completed questionnaires (98% through telephone interviews) on AEFIs in the first 7 days after the vaccine administration (out of 200 distributed questionnaires). Of those 132 were first doses and 25 were booster doses administered.

The median age of the study population was 4.5 years (range 0.29 to 26.8 years), 15% were subjects aged less than 1 year, 39% were aged 1–4 years, 40% were aged 5–14 years and 5% were more than 15 years old; the male-to-female ratio was 1.3 (89 males and 68 females).

The majority of subjects were high-risk individuals (64%); this included those with chronic health conditions. Approximately 25% of these individuals had allergic, 13% neurologic conditions due to previous infectious diseases, 12% neurologic conditions, 11% blood diseases, 6% nephrological conditions and 33% other underlying conditions.

The number of questionnaire performed for the 7-day follow-up was 60 for the first dose and 26 for the second, for 44 completed questionnaires the information on the dose administered was not available; therefore, the follow-up to assess any event within 7 days of vaccination was completed for 83% (*n* = 130) of vaccinated individuals.

Overall, 130 (82.8%) of the vaccinated children reported a total of 311 AEFIs: 147 events (47%) after administration of first dose and 58 (19%) after administration of the booster doses, in 106 (34%) events was not possible identify if were reported after the first or the booster doses (Table 1). The majority of AEFIs reported 7 days from vaccination were of little clinical importance (Table 2) and were concentrated in the first day after vaccine administration (Table 3). No hospitalizations or EDs access were reported.

**Table 1:** Characteristics of vaccinated individuals (*n* = 157) with at least one event within 7 days of vaccination

	Number of subjects vaccinated*			
	Total	With any event (%)		*P* value
Age				
≤ 1	23	17	(14)	*0.300*
1–4	58	49	(40)	
5–14	60	51	(42)	
≥ 15	8	5	(4)	
Sex				
Males	89	73	(56)	*0.767*
Females	68	57	(44)	
High risk condition				
No	56	48	(37)	*0.472*
Yes	101	82	(63)	
Detail of high risk conditions				
Anaphylactic syndromes	25	24	(96)	
Cardiologic diseases	8	6	(75)	
Gastroenterologic diseases	6	4	(67)	
Immunodeficiencies	7	5	(71)	
Neurologic diseases	13	11	(85)	
Previous severe meningitis	13	11	(85)	
Haematologic diseases	11	8	(73)	
Nephrologic diseases	6	3	(50)	
Prematurity	4	4	(100)	
Earing disorders	2	2	(100)	
Others (Genetic, Infectious, Traumatic disorders)	6	4	(96)	

*vaccinated with any dose of vaccine

**Table 2:** Distribution of events within 7 days of vaccination by type of vaccine

Events	Number of vaccinated subjects with one dose	Number of vaccinated subjects with two doses	Total
Local Symptoms			
Local swelling/tenderness	40	12	52
Refusal to move the extremity	28	17	54
Systemic Symptoms			
Persistent, inconsolable crying lasting ≥3 h	37	11	48
Fever ≥37.5 °C	29	13	42
Severe adverse reactions			
Hypotonia	5	3	8
Hypo-responsiveness	4	1	5
Urticaria/angioedema	4	1	5

*^Total events may not equal the sum of individual symptoms reported, as vaccinated subjects were allowed to report multiple symptoms*

**Table 3:** Proportion of AEFIs during the 7 days following vaccination

	Day 1	Day 2	Day 3	Day 4	Day 5	Day 6	Day 7
Tenderness/swelling or erythema							
Absent	59.9	64,3	76,4	86,6	91,1	94,9	96,2
Mild	38.8	34,4	22,9	12,7	8,3	4,5	3,2
Severe	1.3	1,3	0,6	0,6	0,6	0,6	0,6
Refusal to move the extremity							
Absent	58,6	62,4	80,3	90.4	96.2	98.7	98.7
Mild	29,3	29,9	17.8	8.9	3.2	0.6	1.2
Severe	12,1	7,6	1.9	0.6	0.6	0.6	0.0
Unusual crying							
Absent	59.9	77.7	90.5	93.0	95.5	97.4	96.8
Mild	38.9	19.8	8.3	6.4	4.5	2.6	3.2
Severe	1.3	2.6	1.3	0.6	0.0	0.0	0.0
Fever							
Absent	66.2	84.1	96.2	94.9	97.4	97.5	96.8
Mild (< 38.5 °C)	29.9	15.3	3.2	4.5	1.9	1.3	2.6
Severe (> 38.5 °C)	3.8	0.6	0.6	0.6	0.6	1.3	0.6
Hypotonia							
Absent	92.4	96.8	98.7	98.1	99.4	99.4	99.4
Mild	7.0	3.2	1.3	1.9	0.6	0.6	0.6
Severe	0.6	0.0	0.0	0.0	0.0	0.0	0.0
Hypo-responsiveness							
Absent	96.2	98.1	98.1	98.7	0.0	0.0	0.0
Mild	0.3	1.9	1.9	1.3	0.0	0.0	0.0
Severe	0.0	0.0	0.0	0.0	0.0	0.0	0.0
Urticaria/angioedema							
Absent	99.4	98.1	98.1	99.4	0.0	0.0	0.0
Mild	0.6	1.9	1.9	0.6	0.0	0.0	0.0
Severe	0.0	0.0	0.0	0.0	0.0	0.0	0.0

The most frequent AEFIs reported were local symptoms (tenderness, *n* = 76; refusal to move the extremity, *n* = 73), followed by unusual crying (*n* = 67), fever ≥37.5 °C (*n* = 65), and hypotonia and hypo-responsiveness (*n* = 24), with no significant differences for vaccines doses administered (Table 2).

Moreover, the most common collateral effects of the Bexsero vaccine were: tenderness/swelling and erythema of the site of injection (40.12%); unusual crying (40.12%); fever (33.76%); and refusal to move the extremity (41.4%).

The reported events were all mild, and were mostly evident in the first day after the vaccination and decrease gradually until becoming not significant on the 7th day after the vaccination (Table 3).

The only AEFI that has been perceived as severe by a significant number of vaccines’ parents and caregivers was the refusal to move the extremity (described as severe in 12.1% of all the vaccines). We observed a low incidence of high fever (3.82% of all subjects).

Univariate analysis do not show any significant association between vaccination and moderate to severe reactions (Table 4), however in the multivariate logistic regression model, age was associated with the occurrence of a moderate to severe event within 7 day of vaccination (Table 4). Older ages were less associated with moderate to severe reactions compare to younger ages (OR = 0.92, 95%CI 0.86–0.99, *p* = 0.034).

**Table 4:** Variables associated with any event and severe events within 7 days of vaccination among 157 vaccinated subjects: crude and adjusted OR are reported

	OR (95% CI)	Adjusted OR (95% CI)
Age		
≤ 1	1	0.92 (0.86–0.99)
1–4	0.97 (0.36–2.63)	
5–14	0.84 (0.31–2.24)	
≥ 15	0.38 (0.07–2.02)	
Sex		
Males	1	1
Females	0.48 (0.17–1.32)	0.57 (0.29–1.09)
Vaccine dose		
First	1	1
Second	0.84 (0.30–2.35)	0.81 (0.35–1.83)
Risk conditions		
No	1	1
Yes	1.33 (0.43–4.15)	1.09 (0.56–2.12)

*Variables considered in the multivariate model are age at vaccination, sex vaccine dose and presence of risk condition*

*OR: odds ratio; CI: confidence interval*

## Discussion

Our study identified a 7-day reactogenicity profile consistent with earlier clinical trials with the 4CMenB vaccine and large-scale population-based surveillance, that is known to be generally acceptable, with most collateral effects being mild to moderate in severity [8, 11, 15–17].

The reported events were all mild, and were mostly evident in the first day after the vaccination and decrease gradually until becoming not significant on the 7th day after the vaccination. The only AEFI that has been perceived as severe by a significant number of parents and caregivers was the refusal to move the extremity (described as severe in 12.1% of all the vaccinated). Younger ages reported significantly higher rates of moderate to severe events within 7 days of vaccination.

The AEFIs of the Bexsero vaccine was evaluated in a large paediatric study of 7.190 subjects aged 6 weeks or older [18]. Main AEFIs described have been local swelling or erythema for 50% of adults and 80% of adolescents, and local induration for 40–60% of the vaccines regardless of age. Fever has been a relatively common collateral effect in infants and young children and it has been especially evaluated in another study by Novartis of 2.249 infants vaccinated during their second year of life, in which about half have developed a temperature of at least 38 °C [18]. Similarly Gossger et al. have described in a study involving 1.885 infants an incidence of fever of 39 grades or higher in 10 to 15% of subjects given the Bexsero vaccine [8]. Carter’s study of 2013 showed a similar reactogenicity of 4CMenB in infants. Children and adults [19]. Injection-site tenderness/pain, erythema and induration have been the commonest collateral effects reported occurring within 7 days after the vaccination, with an incidence of respectively 55–98%, 41–70% and 40–50% of all vaccines. The most frequent solicited systemic reactions occurring within 7 days of the first 4CMenB dose were irritability (60–79% of subjects), sleepiness (40–73%), change in appetite (32–59%), and unusual crying (28–68%) in infants, and malaise (30–51% of injections), myalgia (28–42%), headache (28%; 42%), arthralgia (19–25%), and nausea (12–16%) in adolescents and adults. During the first 7 days after administration fever > 38 C occurred in 38% of infants who received 4CMenB alone and in 58–61% of those who received 4CMenB concomitantly with the 6-in-1 and PCV7 vaccines. Fever has been a cause of a brief hospitalization for 6 infants in the study [19].

Moreover, a recent active surveillance study assessed AEFIs with acute onset within 7-days post-immunization and impact of antipyretic prophylaxis in approximately 50,000 individuals that received a first and a second dose of 4CMenB [15]. The reported incidence of fever highest in children < 2 years and they registered that among children < 10 years old, ≥2doses of paracetamol prophylaxis significantly reduced fever incidence on days1–2 after dose1&2.

In our study the lower incidence of fever and local reactions in our sample of vaccines could reflect the administration of paracetamol in infants and young infants, that is not recommended by the NIP [5] as a practice. However, this decision was taken based on the particular population we usually vaccinated in our service (subjects at risk).

Considering AEFIs, in our study we identified a very low incidence rate. In particular hypotonia occurred in 5% of all the administered doses, hypo-responsiveness occurred and urticaria/angioedema in 3% of all the administered doses. It is important to emphasize that we did not observe any serious event after the vaccination. In particular, we did not register any case of febrile seizures nor Kawasaki disease in all the vaccines; however, our sample size was very small. None of our patients required hospitalization after the vaccination.

Our data confirm the very low incidence of severe collateral effects of 4CMen B vaccination, as is described in the literature above described. As for other AEFIs, we found also a lower incidence of severe adverse events than that described in other studies. EMA - CHMP in a large paediatric study of 2013 observed 3 cases of febrile seizures occurred within 48 h after vaccination and 6 cases of Kawasaki syndrome [18]. A specialist group considered that 2 of the 6 cases of Kawasaki syndrome were “possibly related” to the meningococcal B vaccine [18].

Our study has several limitations, first the missing data about the immunogenicity of 4CMenB in infants and children affected by chronic medical conditions. Actually, we cannot be sure that the Bexsero vaccine is equally immunogenic in those kind of patients and further studies are needed. Secondly, we used two different systems for data collection (telephone interviews and a web-based system). However, the majority (98%) of parents were contacted by telephone, and, therefore, the risk of an interview bias in data collection was minimal.

From our knowledge this is the first study about 4CMenB administration to infants and children with risk chronic medical conditions. In our sample, we considered each chronic diseases separately to understand the risk factors associated with specific risks’ condition clinicians to pay a particular attention to patients during and after the vaccine administration. In infants born prematurely we didn’t observe apnea, which is believed to be a risk by the Novartis [19]. In patients affected by neurological diseases we didn’t observe a worsening of epilepsy nor any episode of febrile seizure in the 7 days of observation after the vaccination. In patients with cardiologic diseases it was not noted any episode of apnea nor cyanosis. In patients with coagulation disorders we didn’t observe hemorrhagic phenomena.

## Conclusions

We have evaluated reattogenicity to 4CMenB in a group of healthy infants and children and in a group of infants and children affected by chronic medical conditions. For this purpose we used a questionnaire that was administered to the vaccines’ parents or caregivers for the evaluation of the main collateral effects observed in a period of 7 days after the vaccination. Results of our study demonstrated that the Bexsero vaccine is almost well tolerated, with a low incidence of severe collateral effects. Our results showed also that the occurrence of collateral effects is similar in infants and children that are healthy or affected by chronic medical conditions. This finding is really significant because it is known that patients affected by chronic medical conditions are at increased risk of mortality and complication related to invasive meningococcal disease, and the possibility to give them a safe vaccine against Men B establishes an unmissable opportunity of prevention.

## Data Availability

The datasets used and/or analysed during the current study are available from the corresponding author on reasonable request.
